# Supplementary material for: PHA-4/FoxA senses nucleolar stress to regulate lipid accumulation in Caenorhabditis elegans
Source: Nat Commun. 2018 Mar 22;9:1195. doi: 10.1038/s41467-018-03531-2 (PMC5864837; doi:10.1038/s41467-018-03531-2)
Supplement: Supplementary file 1 — Supplementary Information(PDF 1816 kb) [file 41467_2018_3531_MOESM1_ESM.pdf]

Supplementary Information

PHA-4/FoxA Senses Nucleolar Stress to Regulate Lipid Accumulation in *Caenorhabditis elegans*

Wu et al.

Supplementary Figures and Legends

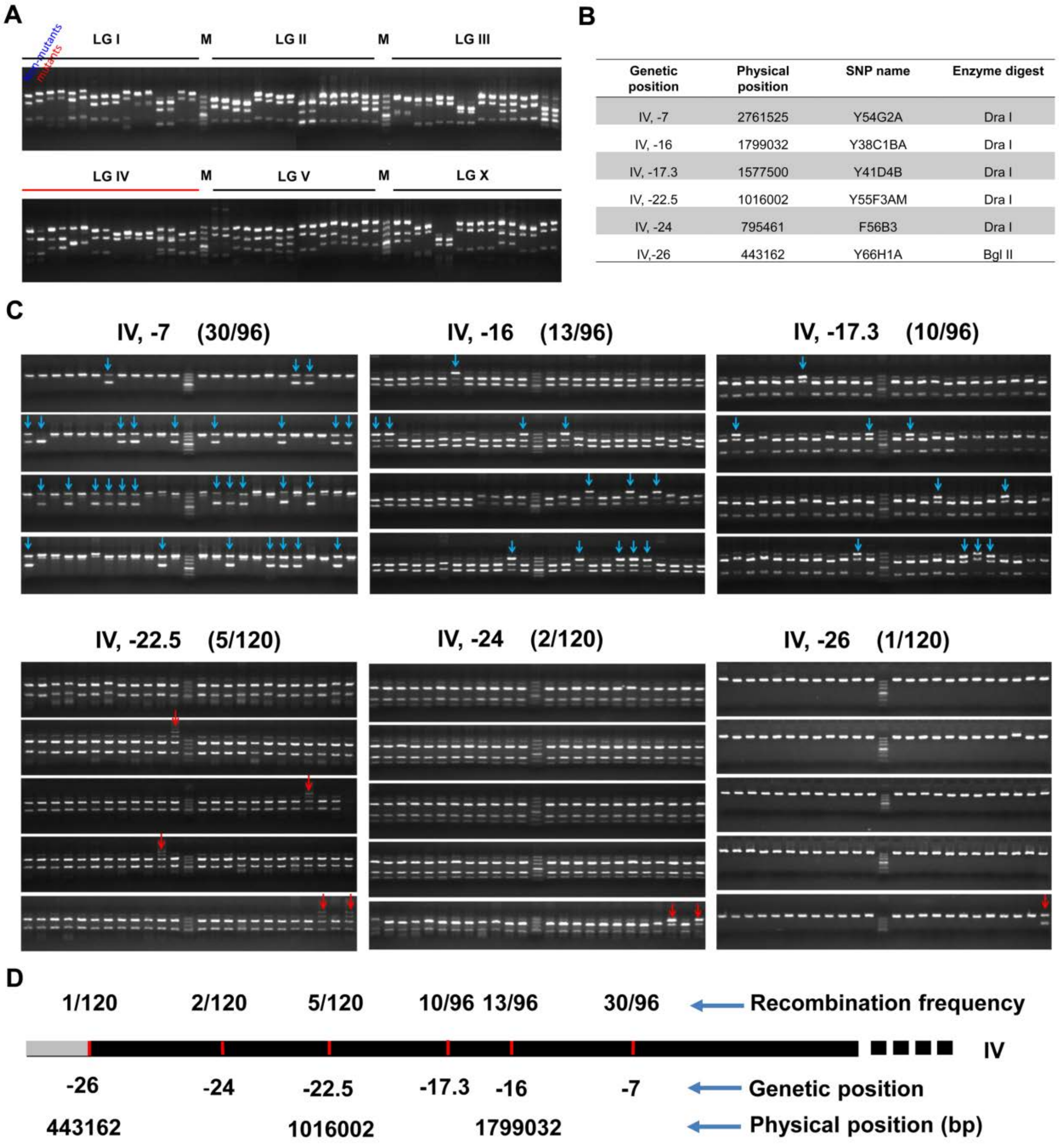

Supplementary Figure 1. Genetic mapping of *kun54* mutation in *C. elegans*.

(A) Initiation chromosome mapping: 48 pairs of primers from the report by Davis *et al*. [1] were used to identify the genotype of either N2 Bristol or CB4856 Hawaiian. Each pair of lanes presents the results obtained for the SNP (single nucleotide polymorphism) at the indicated genetic position, of which odd lanes show the non-mutant populations and even lanes show the mutant populations derived from the cross of *kun54* worms with *CB4856* worms. Linkage shows a tendency toward an

increased proportion of the Bristol N2 genotype compared with the non-mutant lanes for the first SNP on chromosome IV (with red underline). M, 500-bp DNA ladder.

(B and C) Interval mapping: 6 SNP markers selected for interval mapping on chromosome IV are listed in (B). Each lane in (C) is a *kun54* recombinant obtained after crossing with *CB4856* worms. Ninety-six recombinants were used to identify the genotypes of 3 SNPs, Y54G2A, Y38C1BA and Y41D4B (upper panel), and 120 recombinants were used to identify the genotype of another 3 SNPs, Y55F3AM, F56B3 and Y66H1A (lower panel). Most of the recombinants show the N2 Bristol genotype at these SNPs. A portion of the animals show half Bristol and half Hawaiian genotype at one or more loci (indicated by a blue or a red arrow), indicating that crossover occurred at these loci.

(D) Summary of recombination frequency analysis indicates that the mutation site of *kun54* is located in the 0.4-M region on chromosome IV (gray region).

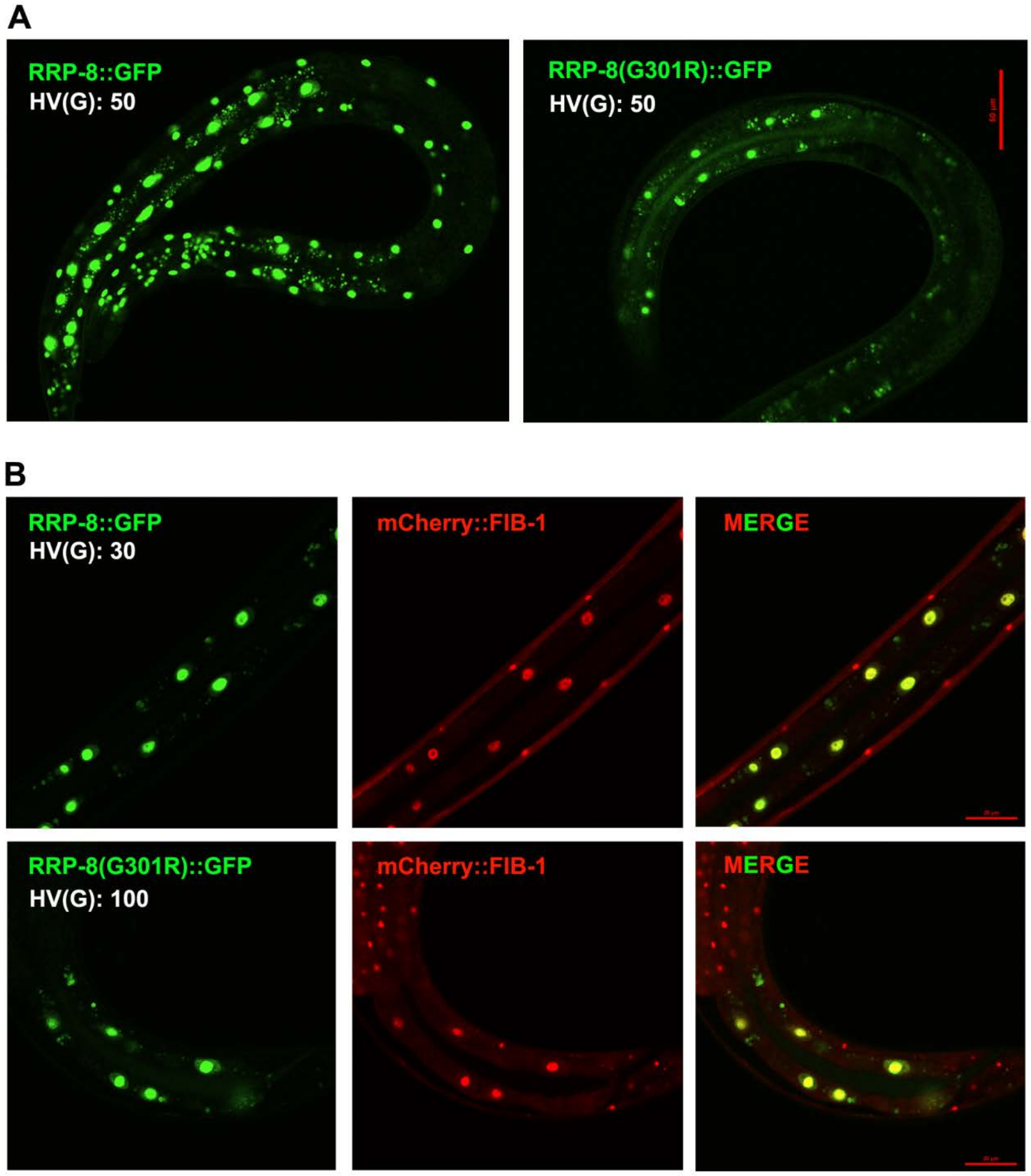

Supplementary Figure 2. Expression of RRP-8 and RRP-8(G301R) in *C. elegans*.

(A) Confocal microscopy of RRP-8::GFP (*WT;kunEx121[Prrp-8::rrp-8::GFP]*) and RRP-8(G301R)::GFP (*WT;kunEx145[Prrp-8::rrp-8(G301R)::GFP]*) fluorescence under the same exposure conditions. Scale bar represents 50 μm.

(B) Confocal microscopy of RRP-8 (G301R)::GFP and RRP-8::GFP fluorescence colocalized with mCherry::FIB-1 under different exposure conditions. HV: Adjustment of detector sensitivity (Voltage). Scale bar represents 20 μm.

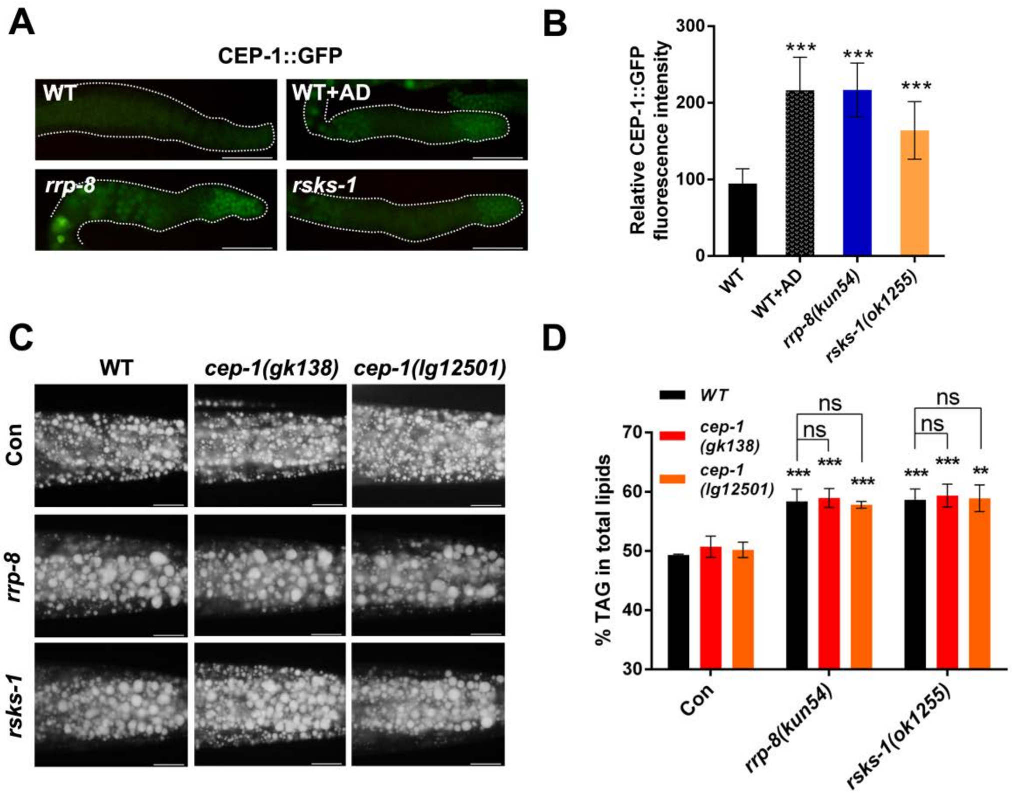

Supplementary Figure 3. CEP-1 is not required for nucleolar stress-induced lipid accumulation.

(A) Fluorescence microscopy of CEP-1::GFP in germline cells of WT, rr*p-8(kun54)*, *rsks-1(ok1255)* and AD-treated worms. Scale bar represents 20 μm.

(B) Relative fluorescence intensity of CEP-1::GFP from (A). Data are presented as the means ± SD of at least 20 worms for each worm strain. Significant difference between WT and a specific worm strain, Student's t-test, ***: P<0.001.

(C) Nile Red staining of fixed worms with different genetic backgrounds. Representative animals; the anterior is indicated on the left, and the posterior is indicated on the right. Scale bar represents 20 μm.

(D) Percentage of triacylglycerol (% TAG) in total lipids (TAG + phospholipids, PL)

analyzed by TLC/GC. Data are presented as the means ± SD of at 4 biological repeats. Significant difference between a specific worm strain and its corresponding control (indicated by the same color), Student's t-test, ***: P<0.001, **: P<0.01. ns: no significant difference.

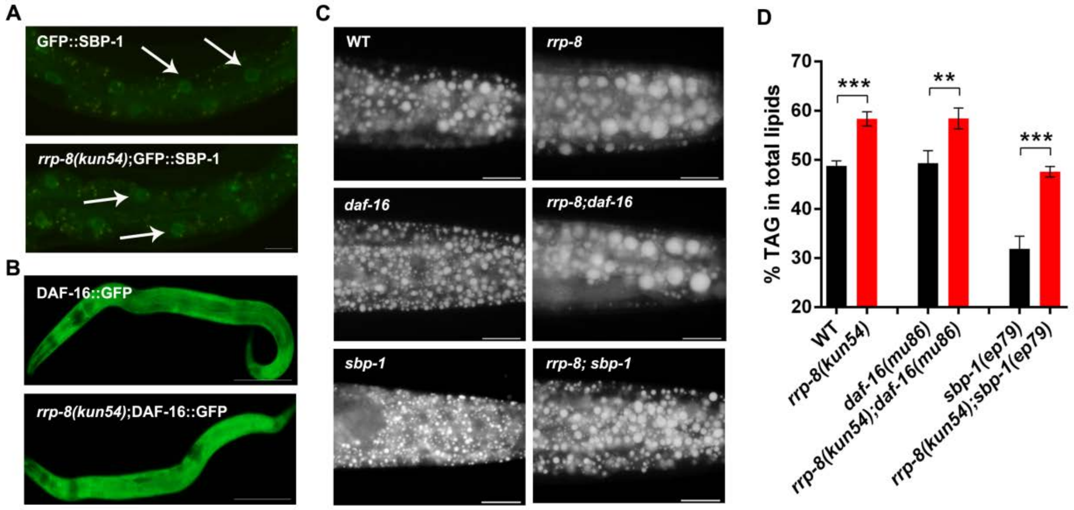

Supplementary Figure 4. Nucleolar stress-induced lipid accumulation is independent of SBP-1 and DAF-16.

(A and B) Fluorescence microscopy of GFP::SBP-1 (*KQ377*) (A) and DAF-16::GFP (*TJ356*) (B) expressing in WT and *rrp-8(kun54)* worms. Representative animals; the anterior is indicated on the left, and the posterior is indicated on the right. Scale bar represents 20 μm in (A) and 100 μm in (B).

(C) Nile Red staining of fixed worms with different genetic backgrounds. Representative animals; the anterior is indicated on the left, and the posterior is indicated on the right. Scale bar represents 20 μm.

(D) Percentage of triacylglycerol (% TAG) in total lipids (TAG + phospholipids, PL)

analyzed by TLC/GC. Data are presented as the means ± SD of at least 3 biological repeats. Significant difference between two indicated worm strains, Student's t-test, ***: P<0.001, **: P<0.01.

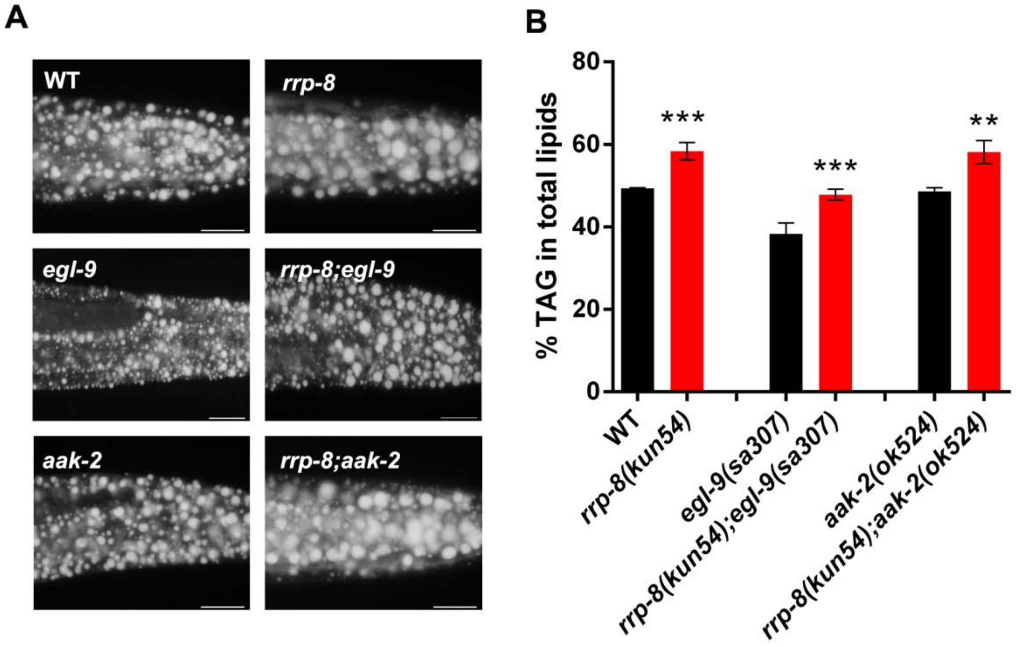

Supplementary Figure 5. EGL-9 and AAK-2 are not required for nucleolar stress induced lipid accumulation.

(A) Nile Red staining of fixed worms. Representative animals; the anterior is indicated on the left, and the posterior is indicated on the right. Scale bar represents 20 μm.

(B) Percentage of triacylglycerol (% TAG) in total lipids (TAG + phospholipids, PL) analyzed by TLC/GC. Data are presented as the means ± SD 3-4 biological repeats. Significant difference between a specific mutant strain with and without the *rrp-8(kun54)* mutation background, Student's t-test, ***: P<0.001, **: P<0.01.

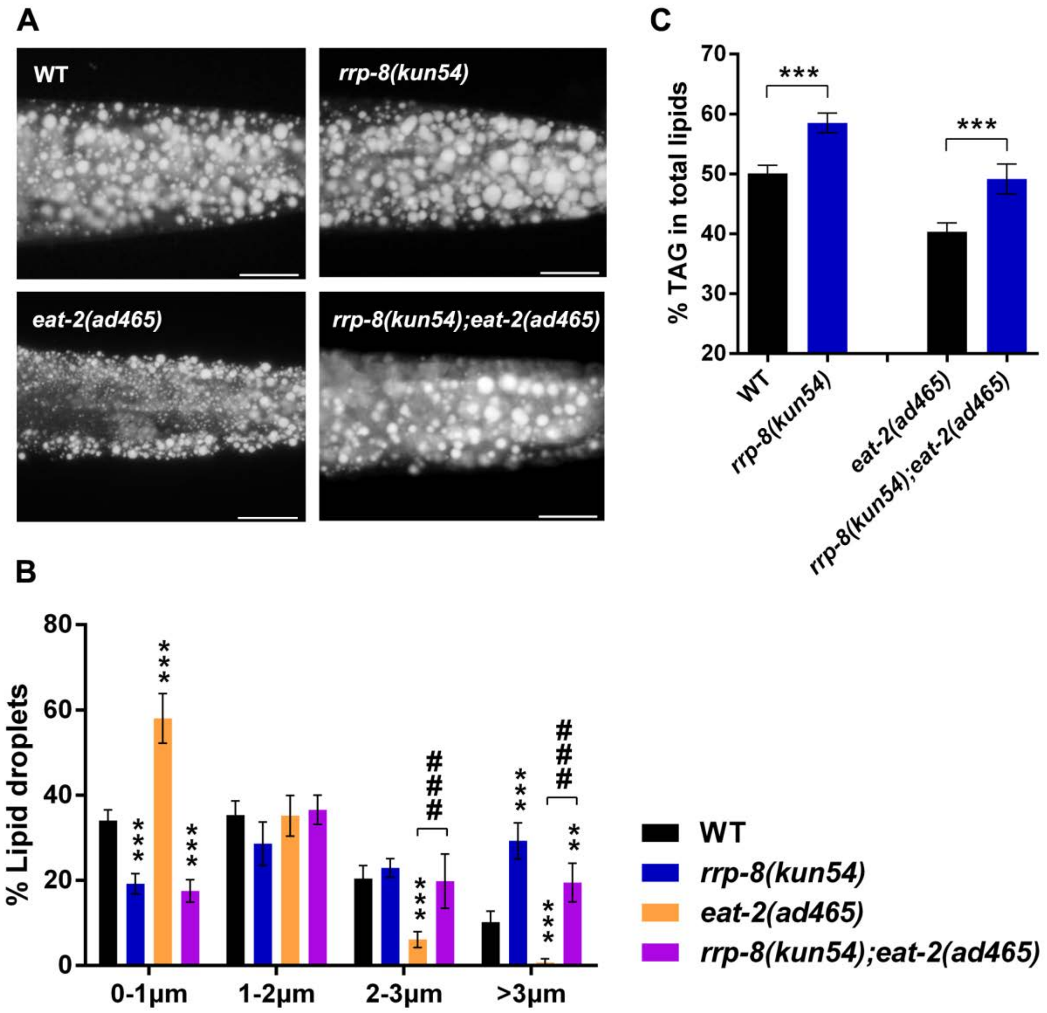

Supplementary Figure 6. The increased lipid accumulation in *rrp-8(kun54)* worms is independent of *eat-2*.

(A) Nile Red staining of fixed worms. Representative animals; the anterior is indicated on the left, and the posterior is indicated on the right. Scale bar represents 20 μm.

(B) Distribution of the lipid droplet size (% lipid droplets) measured by Nile Red staining of fixed worms from (a). Data are presented as the means ± SD of 10 animals for each worm strain. Significant difference between a specific mutant strain and wild

type (WT), Student’s t-test, ***: P<0.001, **: P<0.01. Significant difference between two indicated strains, Student’s t-test, ###: P<0.001.

(C) Percentage of triacylglycerol (% TAG) in total lipids (TAG + phospholipids, PL) analyzed by TLC/GC. Data are presented as the means ± SD of at least 3 biological repeats. Significant difference between two indicated strains, Student’s t-test, ***: P<0.001.

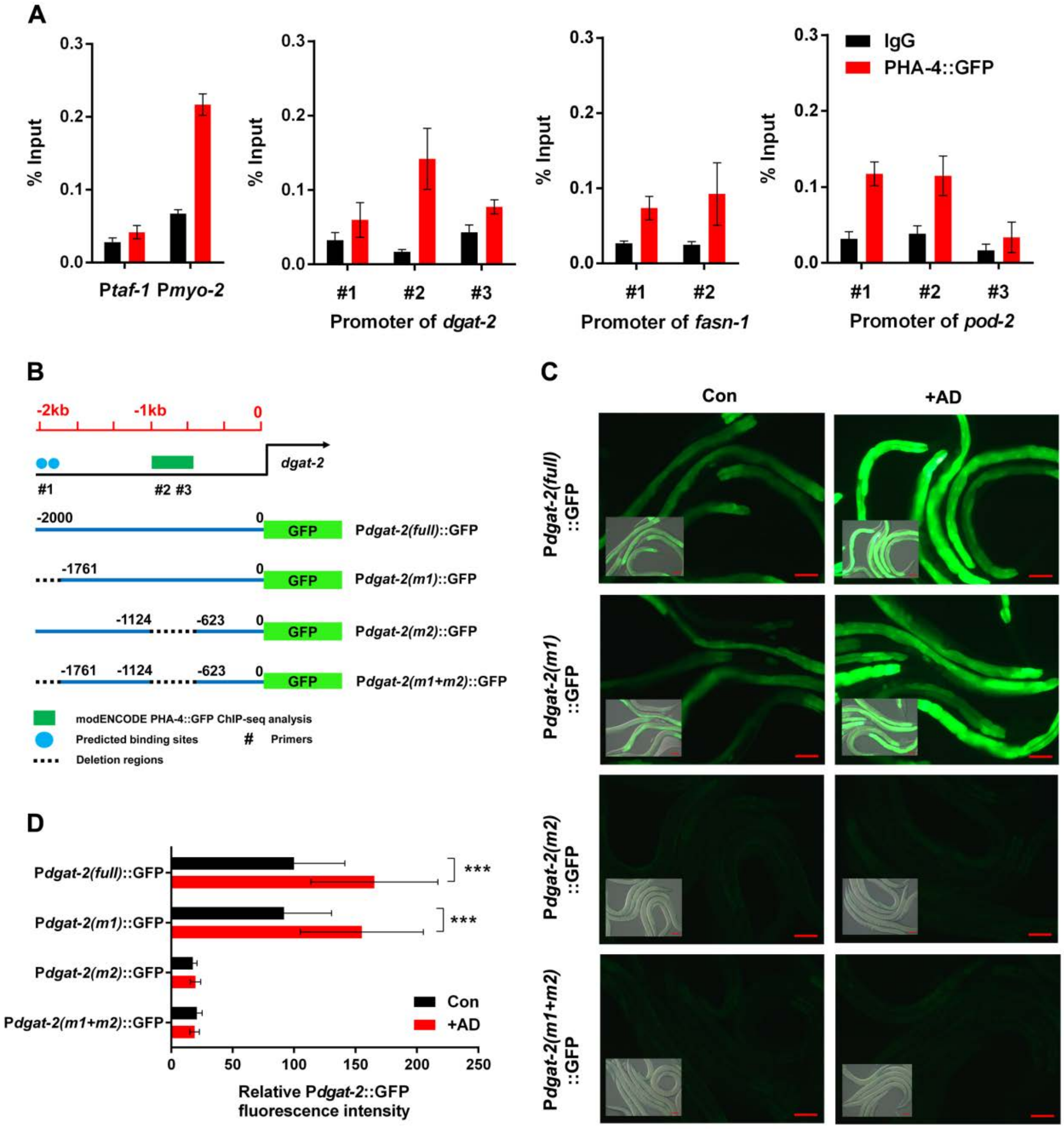

Supplementary Figure 7. PHA-4 bound to the promoters of lipogenic genes.

(A) ChIP analysis of PHA-4 bound to the promoters of *fasn-1*, *pod-2* and *dgat-2* genes. Chromatin extracts of *rrp-8(kun54);wgIs37[pha-4::GFP]* mutant worms were immunoprecipitated with anti-GFP or IgG. In the ChIP-QPCR analysis, the promoter region of *taf-1* and *myo-2* were served as the negative and positive control[2]. Data are presented as the means ± SD of 3 biological repeats.

(B) Schematic diagram of truncated promoter reporter of *dgat-2*. #1, #2 and #3 are three primer pairs that bind to the promoter region of *dgat-2* for ChIP-QPCR analysis

in Figure 6C. P*dgat-2(full)*::GFP indicates the full-length fragment of the *dgat-2* promoter (2 kb upstream from the start codon ATG) fused to GFP as a reporter. P*dgat-2(m1)*::GFP represents a truncated promoter from the fragment -2000 to -1762 of *dgat-2* fused to the GFP reporter. P*dgat-2(m2)*::GFP represents a truncated promoter from the fragment -1123 to -624 of *dgat-2* fused to the GFP reporter. P*dgat-2(m1+m2)*::GFP represents a truncated promoter without the m1 and m2 fragments of *dgat-2* fused to the GFP reporter.

(C) Fluorescence microscopy of full and truncated *dgat-2* promoter reporters. Fluorescence images of one-day-old adults treated or not with actinomycin D (AD). Scale bar represents 50 μm.

(D) Quantification of the relative GFP fluorescence intensity from (C). Data are presented as the means ± SD of more than 100 worms for each worm strain. Significant difference between a specific worm strain with and without AD treatment, Student's t-test, ***: $P<0.001$.

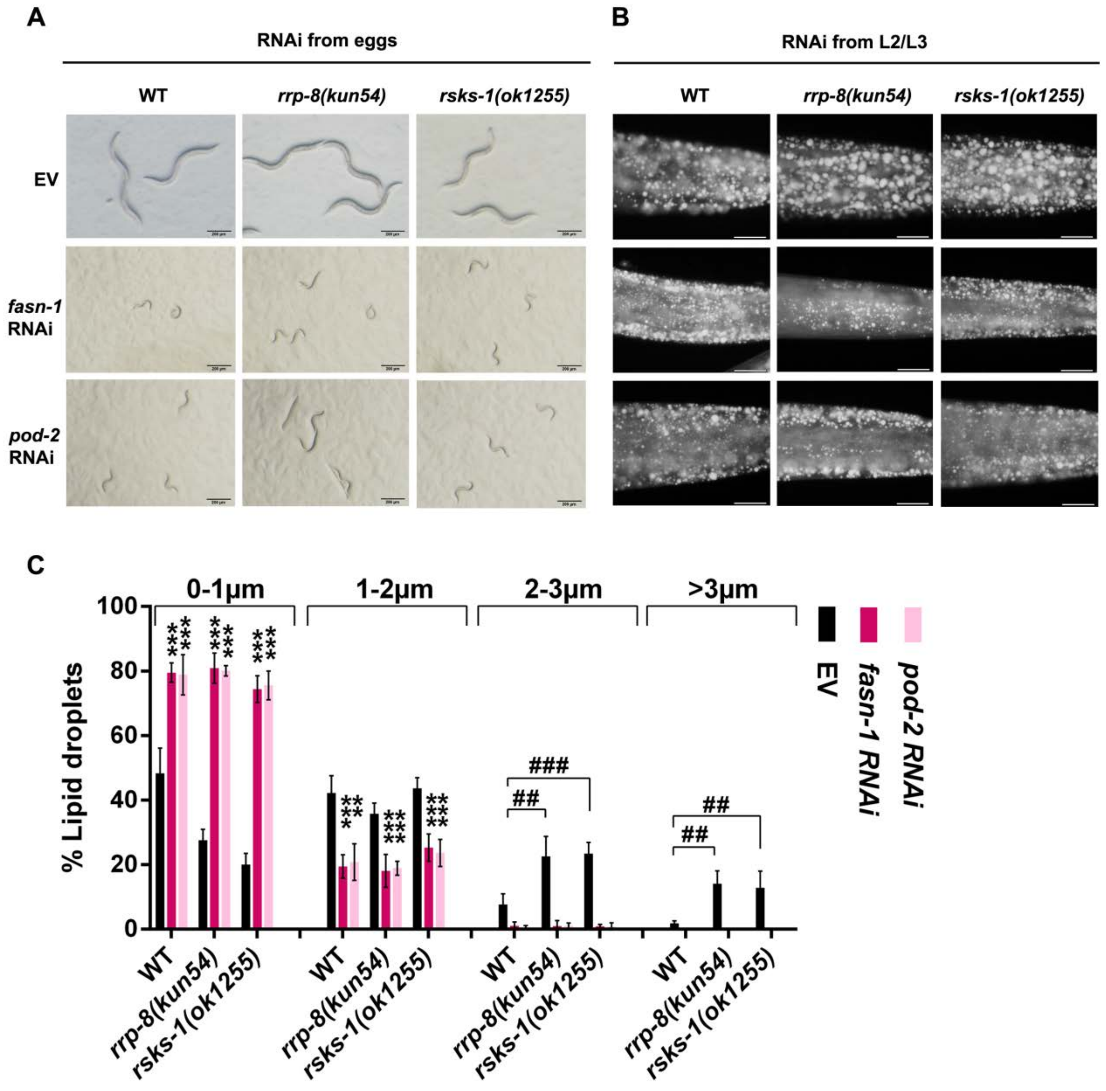

Supplementary Figure 8. RNAi knockdown of *fasn-1* and *pod-2* suppresses nucleolar stress-induced lipid accumulation.

(A) Growth of WT, *rrp-8(kun54)* and *rsks-1(ok1255)* worms treated with *fastn-1* or *pod-2* RNAi from synchronized eggs. EV: empty vector. Scale bar represents 200 μm.

(B) Nile Red staining of fixed worms treated with *fastn-1* or *pod-2* RNAi from the L2/L3 developmental stage. Representative animal, anterior is indicated on the left, and the posterior is indicated on the right. Scale bar represents 20 μm.

(C) Distribution of lipid droplets size (% lipid droplets) measured by the Nile Red staining of fixed worms from (B). Data are presented as the means ± SD of 10

animals for each worm strain. Significant difference between WT and the indicated worm strain, Student's t-test, ###: P<0.001, ##: P<0.01. Significant difference between a specific worm strain with and without RNAi knockdown, Student's t-test, ***: P<0.001, **: P<0.01.

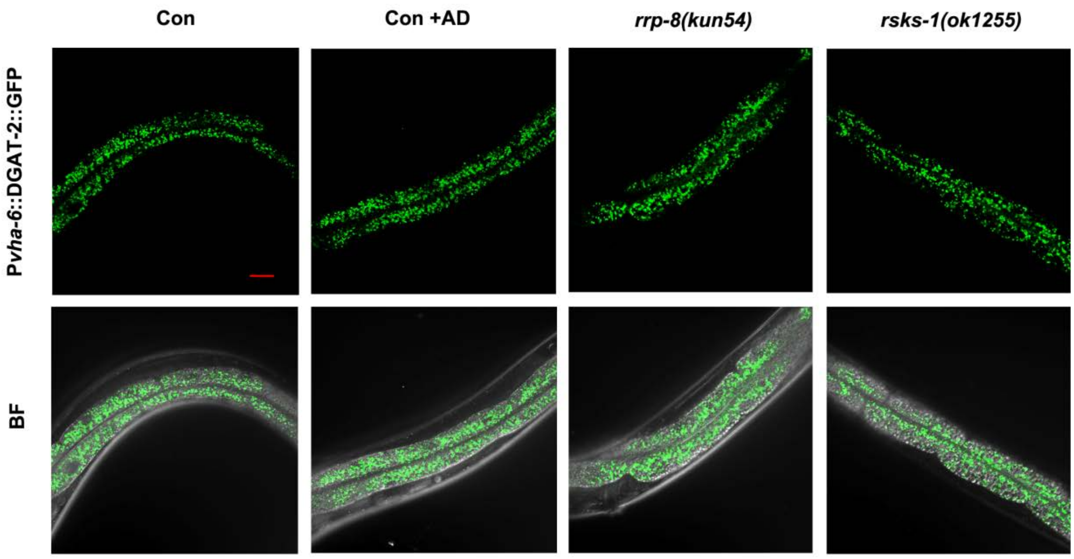

Supplementary Figure 9. Confocal microscopy of P*vha-6*::DGAT-2::GFP [*hjSi56(vha-6p:: 3×FLAG-TEV-GFP::dgat-2::let-858 3'UTR)*] fluorescence in different genetic backgrounds. BF: bright field. Scale bar represents 20 μm.

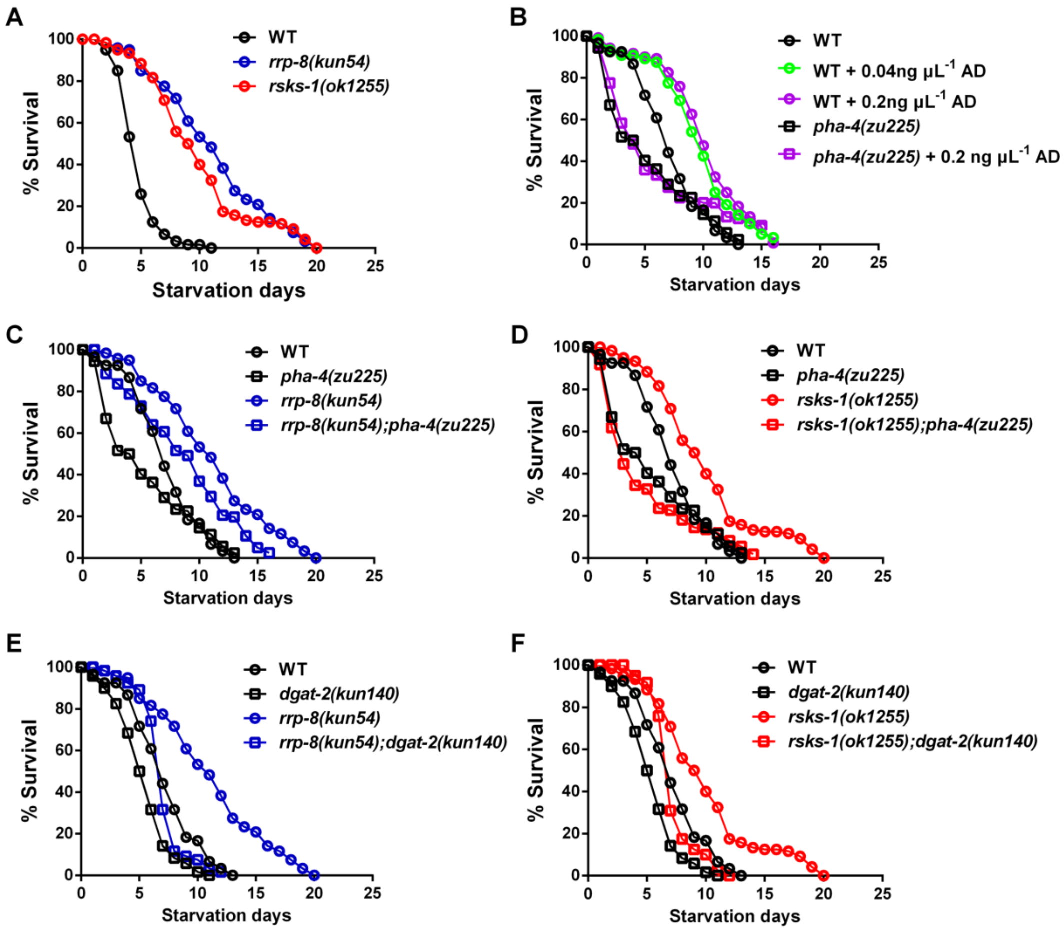

Supplementary Figure 10. Nucleolar stress promotes starvation survival.

(A-F) Starvation survival of WT, *rrp-8(kun54)* and *rsks-1(ok1255)* worms (A); WT and *pha-4(zu225)* worms treated with or without AD (B); *pha-4(zu225)* and *rrp-8(kun54);pha-4(zu225)* worms (C); *pha-4(zu225)* and *rsks-1(ok1255);pha-4(zu225)* worms (D); *dgat-2(kun140)* and *rrp-8(kun54);dgat-2(kun140)* worms (E); as well as *dgat-2(kun140)* and *rsks-1(ok1255);dgat-2(kun140)* worms (F). Synchronized L1 worms were raised on an NGM plate, and grown to L4 stage prior to starvation, and then transferred to M9 medium without food to assay starvation survival. n=120 animals for each worm strain unless specifically indicated (Supplementary Table 1).

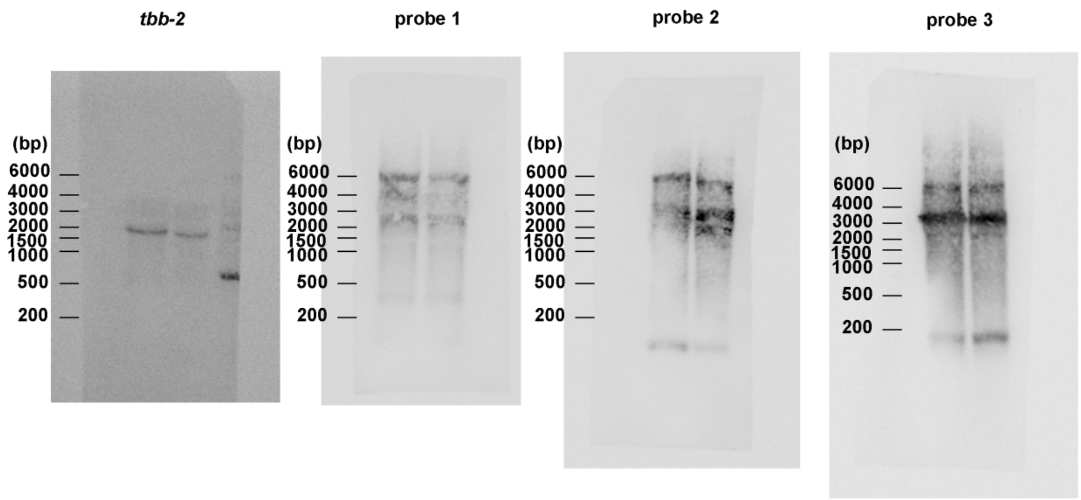

Supplementary Figure 11. Uncropped scans of northern blots.

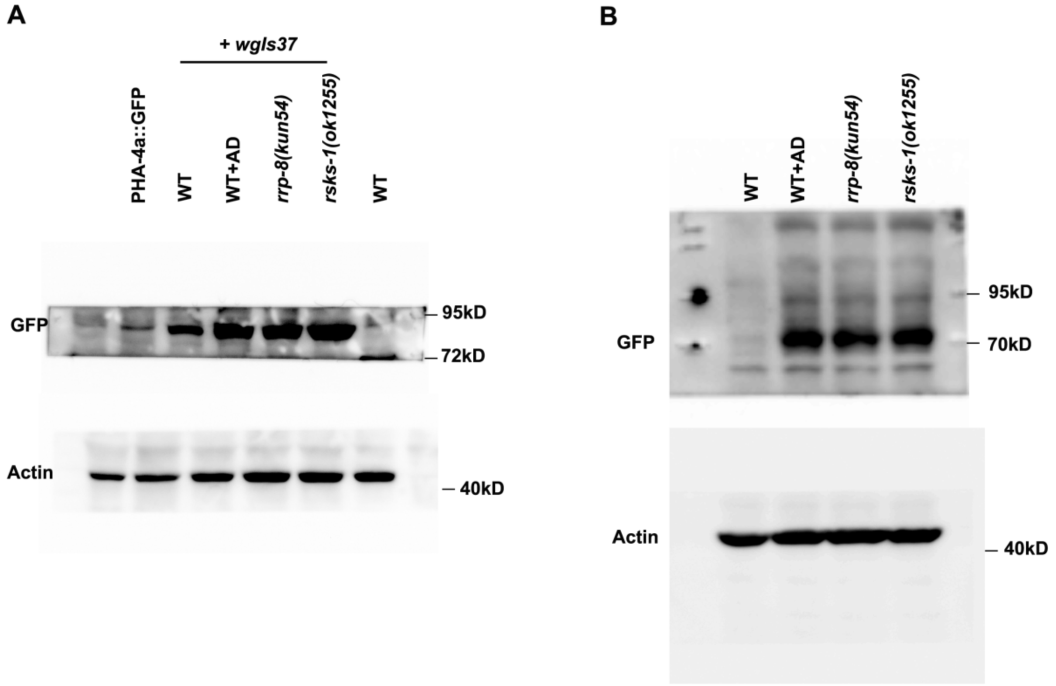

Supplementary Figure 12. Uncropped scans of western blots.

(A) Immunoblotting of PHA-4::GFP (*wgIs37[pha-4::GFP]*) with anti-GFP antibody in WT, *rrp-8(kun54)*, *rsks-1(ok1255)* and AD-treated background worms at one day of adulthood. WT worms without GFP was used as a negative control, and WT worms with PHA-4a::GFP {*kunEx136[Pvha-6::pha-4a::GFP]*} as a positive control. The relative protein levels of PHA-4::GFP were labeled.

(B) Immunoblotting of DGAT-2::GFP. One-day-old WT, *rrp-8(kun54)*, *rsks-1(ok1255)* and AD-treated worms expressing DGAT-2::GFP were harvested for lysing and immunoblotting with anti-GFP antibody.

Supplementary Table 1. Statistical analysis of starvation survival

Strains	Mean survival time ± SD (days)	Median survival time (days)	75 %th (days)	N (4 replicates)	p value
N2 (wild type)	7.44 ± 0.39	7	9	30/30/30/30	-
rrp-8(kun54)	11.14 ± 1.05	11	14	30/30/30/30	0.0006*
rsks-1(ok1255)	10.03 ± 0.95	9.5	12	30/30/30/30	0.0024*
N2 + 0.04 ng μl^{-1} AD	10.70 ± 0.35	11	12.75	30/30/30/30	<0.0001*
N2 + 0.2 ng μl^{-1} AD	11.24 ± 0.84	11	13.75	30/30/30/30	0.0002*
pha-4(zu225)	6.43 ± 0.43	5	9	33/31/30/30	0.0130*
rrp-8(kun54);pha-4(zu225)	9.22 ± 1.04	9	12.25	27/28/29/30	0.0411#
rsks-1(ok1255);pha-4(zu225)	5.88 ± 0.34	4	7	30/30/20/30	0.0002$
pha-4(zu225) + 0.2 ng μl^{-1} AD	7.26 ± 0.76	5	9	30/30/30/30	0.0004†
dgat-2(kun140)	6.48 ± 0.57	6.5	8	30/30/30/30	0.0326*
rrp-8(kun54);dgat-2(kun140)	8.15 ± 0.43	8	9	30/30/30/30	0.0019#
rsks-1(ok1255);dgat-2(kun140)	8.35 ± 0.25	8	9	30/30/30/30	0.0142$

Note: Starvation survival was assayed in different worm strains. p values were calculated by Student's t-test. The symbol * denotes the effect relative to N2 (wild type), # denotes the effect relative to *rrp-8(kun54)*, $ denotes the effect relative to *rsks-1(ok1255)*, † denotes the effect relative to N2 + 0.2 ng μl^{-1}AD.

Supplementary Table 2. Information of worm strains used in this study.

	Strains	Genotype	Source
**Mutants**	CB4856	the polymorphic Hawaiian wild type strain	CGC
	RB754	*aak-2(ok524)*X	CGC
	TJ1	*cep-1(gk138)*I	CGC
	XY1054	*cep-1(lg12501)*I	CGC
	CF1038	*daf-16(mu86)*I	CGC
	BL1049	*dgat-2(kun140)*V	BL
	BL1062	*dgat-2(kun141)*V	BL
	JT307	*egl-9(sa307)*V	CGC
	GC734	*pro-2(na27)*II	CGC
	GC735	*pro-3(ar226)*V	CGC
	PD8120	*smg-1(cc546)*V	CGC
	SM190	*smg-1(cc546);pha-4(zu225)*I	CGC
	BL826	*rrp-8(kun54)*IV	BL
	BL192	*rrp-8(kun122)* IV	BL
	RB1206	*rsks-1(ok1255)*III	CGC
**Transgenic strains**	KQ377	WT;*ftIs7[Psbp-1::gfp::sbp-1]*	CGC
	TG12	*cep-1(lg12501)*I;*unc-119(ed4)*III;*gtIs1[cep-1::gfp,unc-119(+)]*	CGC
	OP37	*unc-119(ed3)*III;*wgIs37[pha-4::TY1::EGFP::3xFLAG,unc-119(+)]*	CGC
	TJ356	WT;*zIs345[daf-16::gfp]*	CGC
	VS29	*hjSi56[vha-6p::3xFLAG::TEV::GFP::dgat-2::let-858 3'UTR]*	CGC
	BL1116	*unc-119(ed3);kunIs148 [Pdgat-2::dgat-2::GFP,unc-119(+)]*	BL
	BL938	WT;*kunEx121[Prrp-8::rrp-8::GFP]*	BL
	BL1113	WT;*kunEx145[Prrp-8::rrp-8(G301R)::GFP]*	BL
	BL1014	*unc-119(ed3);kunIs124[Prrp-8::rrp-8::gfp,unc-119(+)],*	BL
	BL1058	*kunEx136[Pvha-6::pha-4a::GFP],*	BL
	BL1059	*kunEx137[Pvha-6::pha-4b::GFP]*	BL
	BL1060	*kunEx138[Pvha-6::pha-4c::GFP]*	BL
	SHG388	*ustIs36[mcherry::fib-1]*	Shouhong Guang lab
	BL1476	*unc-119(ed3);kunEx204[Pdgat-2(full):: GFP, unc-119(+)]*	BL
	BL1477	*unc-119(ed3);kunEx205[Pdgat-2(m1):: GFP, unc-119(+)]*	BL

BL1478	*unc-119(ed3);kunEx206[Pdgat-2(m2):: GFP, unc-119(+)]*	BL
BL1479	*unc-119(ed3);kunEx207[Pdgat-2(m1+m2):: GFP, unc-119(+)]*	BL

Note: BL indicates that the strain was created in Bin Liang's laboratory.

Supplementary Table 3. Sequence information of primers used in this study

Primer name	sequences	Note
**Cas-9 knock out**		
sg-rrp-8 F	GGAAGCAGATCCAATTGCAGGTTTTAGAGCTAGAAATAGC	*rrp-8(kun122)*
sg-rrp-8 R	CTGCAATTGGATCTGCTTCCAAACATTTAGATTTGCAATTC	
sg-dgat-2 F1	GGGTGAACATCTTCTCCCCAGTTTTAGAGCTAGAAATAGC	*Dgat-2(kun140) and dgat-2(kun141)*
sg- dgat-2 R1	TGGGGAGAAGATGTTCACCCAAACATTTAGATTTGCAATTC	
sg- dgat-2 F2	GTCGCCTTGGTCGTCTGGTTGTTTTAGAGCTAGAAATAGC	
sg- dgat-2 R2	AACCAGACGACCAAGGCGACAAACATTTAGATTTGCAATTC	
sg- dgat-2 F3	GTGAGAGAAGCCGTGAAGACGTTTTAGAGCTAGAAATAGC	
sg- dgat-2 R3	GTCTTCACGGCTTCTCTCACAAACATTTAGATTTGCAATTC	
sg- dgat-2 F4	GGTCGGAGCGCCTATTCAAGGTTTTAGAGCTAGAAATAGC	
sg- dgat-2 R4	CTTGAATAGGCGCTCCGACCAAACATTTAGATTTGCAATTC	
**Transgene construct**		
c-pCFJ151 F	AGATACCTAGGTGAGCTCTGG	
c-pCFJ151 R	TTCGTGGATCCAGATATCC	
dgat-2 F	GGATATCTGGATCCACGAAgctcgtaaattaaaatgccg	*kunIs143[Pdga-2::dgat-2::gfp, unc-119(+)]*
dgat-2 R	GTCGACCTGCAGGCATGCAACTGAAAAACGAGCCGAGTGTC	
GFP+unc-54 3’UTR F	TTGCATGCCTGCAGGTCGAC	
GFP+unc-54 3’UTR R	CCAGAGCTCACCTAGGTATCTGCCGACTAGTAGGAAACAGT	
rrp-8-gfp F	GGATATCTGGATCCACGAAcgcaattgcacactttagg	*kunIs124[Prrp-8::rrp-8::gfp, unc-119(+)]*
rrp-8-gfp R	GTCGACCTGCAGGCATGCAATAGGAAACAGTTATGTTTGG	
rrp-8(G301R)-gfp F	CAAAAATGAGATTCGAGCAATCACACCGTCGTGAGCTAAC	*kunEx145[Prrp-8::rrp-8(G301R) ::GFP]*
rrp-8(G301R)-gfp R	TTGCTCGAATCTCATTTTTGTGATCGCCTCGCAAAATTG	
Pvha-6 F	GCAGGTCGACTCTAGAGGATTAGAGCATGTACCTTTATAGG	
Pvha-6 R	GTAGGTTTTAGTCGCCCTG	*kunEx136[Pvha-6::pha-4a::gfp], kunEx136[Pvha-6::pha-4a::gfp], kunEx136[Pvha-6::pha-4a::gfp].*
pha-4a(cds) F1	CAGGGCGACTAAAACCTACATGACATCGCCATCCAGTGATG	
pha-4b(cds) F2	CAGGGCGACTAAAACCTACATGTTGCCAAATGGAACCAC	
pha-4c(cds) F3	CAGGGCGACTAAAACCTACATGAACGCTCAGGACTATCTG	
pha-4 (cds) R	GGTACCCTCCAAGGGTCCTCTAGGTTGGCGGCCGAGTTCG	
**ChIP-QPCR primers**		product size
ChIP-q-PCR Ptaf-1 F	ttggttgaggctcacactac	101 bp
ChIP-q-PCR Ptaf-1 R	GGTTGAGGCTCACACTACAA	

ChIP-q-PCR Pmyo-2 F	caagataggttggtaatcgc	92 bp
ChIP-q-PCR Pmyo-2 R	CAATGAGTACAGTGTGTGCG	
ChIP-q-Pfasn-1 F1	ggaaagcagcgataagccac	119 bp
ChIP-q-Pfasn-1 R1	TCTGCTACTGGCATAATCTC	
ChIP-q-Pfasn-1 F2	ttcttgtgtagtccatgtcc	114 bp
ChIP-q-Pfasn-1 R2	ACTAGAAGCAGAATTTAGCT	
ChIP-q-Pfasn-1 F3	gaacgtagctgacctctatg	101 bp
ChIP-q-Pfasn-1 R3	TGCCTCTACCGTACTCATAG	
ChIP-q-Ppod-2 F1	acttttccgccttaaaagcg	86 bp
ChIP-q-Ppod-2 R1	AACTGCCGGAAACCTGTGT	
ChIP-q-Ppod-2 F2	agcagcagaggaaccagcat	125 bp
ChIP-q-Ppod-2 R2	GCTCTACCGCTAATTCATTG	
ChIP-q-Ppod-2 F3	aatattgacccttccatagg	124 bp
ChIP-q-Ppod-2 R3	GGTTTTTTCACAGAAATTTCTG	
ChIP-q-Pdgat-2 F1	gctgactacagtactccaac	128 bp
ChIP-q-Pdgat-2 R1	AGTGCACATTCGGCAGAAAT	
ChIP-q-Pdgat-2 F2	tatactttgcctgaaccatg	118 bp
ChIP-q-Pdgat-2 R2	CCACTATTAGGGTGTTCAG	
ChIP-q-Pdgat-2 F3	gtacacggaggctaatggtg	146 bp
ChIP-q-Pdgat-2 R3	AATGTAGAAAGTCACGCT	
**Probes for northern**		product size
Probe 1F	aatactccctccccgcacactcctatatgt	98 bp
Probe 1R	TAATACGACTCACTATAGGGCACACACTACCATCACCAAC	
Probe 2F	cgttgttcacgagtcgtctc	100 bp
Probe 2R	TAATACGACTCACTATAGGGATCGTCAACTAACTTCCTCTC	
Probe 3F	gtcttcggcttgtcgggcaa	85 bp
Probe 3R	TAATACGACTCACTATAGGGACTTGTGATGCTTCTGGACT	
probe-*tbb*-2 F	AGCCAACAACGGAAAGTATG	110 bp
probe-*tbb*-2 R	TAATACGACTCACTATAGGGAGTTGTCAGGACGGAACAGC	

Supplementary References:

1. Davis, M.W. *et al.* Rapid single nucleotide polymorphism mapping in C. elegans. *BMC genomics* **6**, 118 (2005).
2. Hsu, H.T. *et al.* TRANSCRIPTION. Recruitment of RNA polymerase II by the pioneer transcription factor PHA-4. *Science (New York, N.Y.)* **348**, 1372-1376 (2015).
