# Supplementary material for: PHA-4/FoxA senses nucleolar stress to regulate lipid accumulation in Caenorhabditis elegans
Source: Nat Commun. 2018 Mar 22;9:1195. doi: 10.1038/s41467-018-03531-2 (PMC5864837; doi:10.1038/s41467-018-03531-2)
Supplement: Supplementary file 3 — Description of Additional Supplementary Files(PDF 165 kb) [file 41467_2018_3531_MOESM3_ESM.pdf]

**Description of Additional Supplementary Files**

File Name: Supplementary Data 1
Description: Information of worm strains used in this study.

File Name: Supplementary Data 2
Description: Sequence information of primers used in this study.
